# The Gut Microbiota: An Essential Component in Understanding Pediatric Obesity: A Narrative Review

**DOI:** 10.3390/nu18060952

**Published:** 2026-03-18

**Authors:** Vasile Valeriu Lupu, Alin Horatiu Nedelcu, Reka Borka-Balas, Carmen Rodica Anton, Irina Tarnita, Alice Azoicai, Lorenza Forna, Dragos Munteanu, Sorana Caterina Anton, Shwan Karwan Shawais, Minerva Codruta Badescu, Delia Lidia Salaru, Ionela Daniela Morariu, Emil Anton, Florin Petrariu, Ancuta Lupu

**Affiliations:** 1 Grigore T. Popa University of Medicine and Pharmacy, 700115 Iasi, Romania; vasile.lupu@umfiasi.ro (V.V.L.); alin.nedelcu@umfiasi.ro (A.H.N.); carmen.anton@umfiasi.ro (C.R.A.); alice.azoicai@umfiasi.ro (A.A.); lorenza.forna@umfiasi.ro (L.F.); dragos.munteanu@umfiasi.ro (D.M.); sorana.anton@umfiasi.ro (S.C.A.); shwansaddik@gmail.com (S.K.S.); minerva.badescu@umfiasi.ro (M.C.B.); delia.salaru@umfiasi.ro (D.L.S.); ionela.morariu@umfiasi.ro (I.D.M.); emil.anton@umfiasi.ro (E.A.); florin.petrariu@umfiasi.ro (F.P.); ancuta.ignat1@umfiasi.ro (A.L.); 2 Department of Pediatrics, “George Emil Palade” University of Medicine, Pharmacy, Science and Technology, 540142 Targu Mures, Romania; rekaborkabalas@gmail.com

**Keywords:** pediatric obesity, gut microbiota, bacteria, dysbiosis, probiotics

## Abstract

**Background**: Childhood obesity has become a major public health concern worldwide. Increasing evidence suggests that alterations in the gut microbiome may play a significant role in the development and progression of pediatric obesity. This narrative review synthesizes and analyzes recent studies investigating microbiome alterations in children with obesity, highlighting emerging insights and their potential implications for disease management. Understanding the relationship between gut microbial composition and obesity may provide new perspectives for prevention and therapeutic strategies in overweight pediatric populations. This narrative review was conducted through a search of major biomedical databases, including PubMed and Web of Science, complemented by manual screening of reference lists of relevant articles. **Key findings**: Children affected by obesity exhibit significant changes in gut microbiome composition, characterized by reduced microbial diversity and predominance of the Firmicutes and Bacteroidetes phyla. The balance between these two bacterial groups appears critical for maintaining gut homeostasis. Studies consistently report an increased *Firmicutes*-to-*Bacteroidetes* ratio in children with elevated body weight, suggesting that disruption of this balance may contribute to metabolic dysregulation and obesity-related pathologies. Given the essential role of the gut microbiota in nutrient metabolism and energy extraction, dysbiosis in obesity is associated with enhanced energy harvest and lipid absorption. Certain bacterial populations may promote increased caloric uptake, thereby contributing to weight gain and adiposity. Multidimensional interventions, including dietary modification and physical activity, have demonstrated the potential to reduce obesogenic microbiota patterns and restore microbial diversity. Additionally, probiotic supplementation is being investigated as a strategy to reestablish microbial homeostasis and potentially support body mass index reduction. Despite promising findings, further research is required to clarify mechanisms, establish causality, and determine the clinical effectiveness of microbiome-targeted therapies before they can be fully integrated into the management of pediatric obesity.

## 1. Introduction

Obesity is one of the main public health concerns and its prevalence is increasing. By 2030, it is estimated that 20% of adults will be obese and 38% will be overweight on a global scale [1].

Pediatric obesity is also a global health problem. Over the last two decades, the number of cases has increased while the age at diagnosis has decreased [2]. Pediatric obesity is associated with hypertension, type 2 diabetes, dyslipidemia, psychological problems, and fatty liver disease which can continue into adulthood. The gut microbiota influences many biochemical processes, and certain bacterial species can contribute to obesity by regulating lipopolysaccharide (LPS) levels. They promote fat storage and provide extra calories to the host, thereby facilitating weight gain [3,4].

Approximately 10^14^ microorganisms reside within the gastrointestinal tract, playing essential roles in intestinal development, homeostasis, and defense against pathogens. The gut microbiota consists of a diverse community of bacteria, yeasts, and viruses, closely linked to metabolic and immunomodulatory processes. The predominant bacterial phyla in the intestine include *Bacteroidetes*, *Actinobacteria*, *Firmicutes*, *Proteobacteria*, *Fusobacteria*, and *Verrucomicrobia*. Notably, *Firmicutes* and *Bacteroidetes* account for nearly 90% of the gut microbiota composition [5,6].

The gut microbiota plays a key role in nutrient acquisition and energy extraction from the food, influencing energy balance. These mechanisms may serve as fundamental factors in the pathophysiology of obesity [7,8,9,10,11]. In some studies, the researchers transferred the altered microbiota from overweight patients into lean animals and they concluded that, as a result of this inoculation, the animals developed an obese phenotype [12,13]. The microbiome is a dynamic and adaptable component of the human body, influenced by various environmental factors. In particular, the gut microbiota plays a crucial role in pediatric obesity, impacting several metabolic pathways [14].

This malleable ecosystem can shift in response to diet, lifestyle, and external stimuli, affecting processes like fat storage, energy regulation, and inflammation, which are closely linked to the development of obesity in children. In the pediatric population, an imbalance in gut bacteria—known as dysbiosis—can contribute to the development and persistence of obesity. Key differences observed in overweight children include a greater prevalence of pro-inflammatory bacteria and a decrease in beneficial microbial diversity. This imbalance may enhance caloric harvest from the diet, promote low-grade inflammation, and disrupt normal lipid storage mechanisms, ultimately contributing to excessive weight gain and metabolic dysfunction. In addition, early-life factors such as antibiotic exposure and dietary patterns play a pivotal role in shaping gut microbiota composition, potentially increasing the risk of obesity later in childhood. Interventions targeting the microbiota, such as probiotics, prebiotics, and dietary changes, are being explored as potential strategies to mitigate obesity and related metabolic conditions [15,16,17,18,19].

Another key factor in childhood obesity, oxidative stress, can play a significant role in the development of metabolic and cardiovascular diseases. Obesity can lead to heightened levels of free radicals in the body due to factors such as excess fatty tissue, inflammation, and the metabolic activity associated with stored fats. Excess body fat, particularly visceral fat, produces increased amounts of reactive oxygen species as it is metabolically active. This leads to an overproduction of free radicals that can overwhelm antioxidant defenses. Obesity also triggers an inflammatory response in the body, characterized by the release of pro-inflammatory cytokines such as TNF-alpha and IL-6 from adipose tissue. This inflammation can promote oxidative stress and further damage tissues [20,21].

Despite the growing body of literature linking gut microbiota to obesity, significant gaps remain in understanding its specific role in pediatric populations. Current evidence is largely derived from adult studies, and the causative versus consequential nature of microbiota alterations in childhood obesity remains unclear. Moreover, early-life microbial programming, microbial metabolite activity, and the identification of a consistent obesogenic microbial signature in children are not fully elucidated. Therapeutic strategies aimed at modulating the microbiota, including probiotics and lifestyle interventions, show promise; however, pediatric-specific data regarding their long-term efficacy and safety are still limited. Therefore, a comprehensive synthesis of recent findings is necessary to clarify these aspects and to outline future directions for microbiota-targeted interventions in pediatric obesity management.

This narrative review was conducted through a structured search of major biomedical databases, including PubMed and Web of Science, complemented by manual screening of reference lists of relevant articles. The search strategy combined keywords related to gut microbiota, pediatric obesity, short-chain fatty acids, diet, metabolic syndrome, and microbiota-targeted interventions. Eligible studies included original research articles, randomized controlled trials, cohort studies, and systematic reviews published in English that examined associations between gut microbiota and obesity or related metabolic outcomes, particularly in pediatric populations. Data extraction involved collection of information on study design, population characteristics, microbiome assessment methods, and principal metabolic outcomes.

## 2. The Microbial Colonization of the Gut in Infancy and Its Importance

For a long time, it was believed that infant gut colonization begins at delivery. However, recent studies have demonstrated the presence of microbial communities in the meconium [22,23]. In a study conducted under sterile conditions, 320 placental specimens were collected which revealed a unique placental microbiome. Furthermore, researchers established a connection between the placental microbiome and the oral microbiome [24].

Additionally, the study found that maternal probiotic supplementation may influence the expression of genes related to Toll-like receptors in both the placenta and the fetal intestine. This suggests that fetal intestinal immune gene expression can be modified by such supplementation, potentially impacting the development of the fetal immune system [25]. Similarities between the microbiota of the placenta, amniotic fluid, and infant meconium support the hypothesis of microbial prenatal transfer from mother to fetus [26].

Moreover, several other factors influence early intestinal colonization, with gestational age being a key determinant [26]. Studies have shown significant differences between the microbiota of preterm and term infants. The gut microbiota of preterm infants appears to be dominated by *Enterococcus*, *Staphylococcus*, and *Enterobacter* [27,28,29].

The infant’s diet is also a key factor that influences intestinal microbiota. Breastfed infants tend to have a microbiota that is enriched in beneficial bacteria like *Bifidobacterium*, *Lactobacillus*, and *Staphylococcus*, which support healthy gut development and immune function. In contrast, formula-fed infants typically have gut microbiota dominated by bacteria such as *Clostridium*, *Anaerostipes*, and *Roseburia*, which may contribute to different metabolic profiles. This variation is largely due to the unique composition of breast milk, which contains oligosaccharides that selectively promote the growth of beneficial bacteria, while formula lacks these specific nutrients [30].

During the first year of life, the neonatal microbiome undergoes significant development and maturation, gradually evolving into a more complex structure that increasingly resembles the adult microbiome. This process is characterized by an enrichment in bacterial groups such as *Bacteroides* and *Firmicutes* [30,31]. Before the introduction of solid foods, the infant’s microbiome already contains a variety of bacterial genes involved in the metabolism of plant polysaccharides, priming the gut for the digestion of more complex carbohydrates once solid foods are introduced into the diet. Once solid foods are introduced, the gut microbiota becomes gradually enriched in *Bacteroidetes*. In addition, the fecal short-chain fatty acid (SCFA) levels and the expression of genes relevant for carbohydrate metabolism increase [32]. Meanwhile, some studies showed that, from 3 years old, children have an adult-like microbiota [30,31,32,33,34].

A long-term study involving over 900 infants examined the relationship between maternal pre-pregnancy overweight and the likelihood of children being overweight at 1 and 3 years of age. It appears that the microbiota in overweight infants was enriched in members of the *Lachnospiraceae* family [35]. Moreover, there is evidence to suggest that increased levels of *Staphylococcus aureus* and *Bifidobacterium* spp. in infant microbiota is associated with an increased risk of overweight status by age 7 [36]. Exposure to antibiotics during early infancy also significantly impacts the risk of developing obesity. Cho et al. [37] administered low doses of antibiotics to young mice and observed an increase in metabolic hormones, SCFAs, and adiposity levels. Additionally, they reported significant alterations in hepatic lipid and cholesterol metabolism.

Research indicates that the microbiota associated with macrosomic newborns (those born with a birth weight of ≥4000 g) differs significantly from that of newborns with normal birth weight. Specifically, studies have identified a distinct placental microbiota profile in macrosomic infants, which is associated with various maternal and infant clinical characteristics. Key findings reveal variations in the relative abundance of microbial communities across different taxonomic levels, including specific operational taxonomic units, phyla, and genera. This study [38] explored whether certain placental microbiota structures are associated with fetal macrosomia. The researchers compared the relative abundance of microbiota at the level of phylum, family, and genus between macrosomic and control groups. They found a significant increase in the abundance of phyla *Proteobacteria*, *Firmicutes*, and *Gemmatimonadetes*, while unclassified bacteria were significantly less abundant in the macrosomia group than in the control group. At the level of family, the proportions of *Alcaligenaceae* and *Lachnospiraceae* were elevated, and unclassified bacteria were less abundant in the macrosomia group. Genus-level analysis revealed significant differences in twelve genera between the groups. Specifically, *Novosphingobium*, *Achromobacter*, *Acinetobacter*, *Paracoccus*, *Pseudonocardia*, *Woodsholea*, *Nitratireductor*, *Pelomonas*, and unclassified *Alcaligenaceae* were more abundant in the macrosomia group, whereas the *Prevotellaceae*_NK3B31_group, and unclassified bacteria were significantly less abundant compared to the control group. The study suggests that the distinct microbial profiles found in macrosomic newborns may be linked to their metabolic and developmental characteristics, as well as maternal health during pregnancy. This highlights the possible influence of placental microbiota on newborn health and underscores the need for additional research to fully understand these connections [38].

## 3. Microbiome Implications in Pediatric Obesity Occurrence

The gut microbiota is characterized by the presence of genes encoding digestive enzymes that are not found in human cells. These genes play a crucial role in food digestion, contributing to the fermentation and metabolism of various dietary compounds. The primary metabolic byproducts of undigested carbohydrate fermentation are SCFAs, including propionate, butyrate, and predominantly acetate. These SCFAs are produced through the anaerobic fermentation of undigested carbohydrates [39,40].

Beyond their role in digestion, gut microbiota is also involved in the metabolism of pharmacologically active substances, such as phytoestrogens [41]. An imbalance in gut microbiota composition, known as dysbiosis, has been associated with various health conditions, including diabetes and obesity [42].

**Figure 1:**
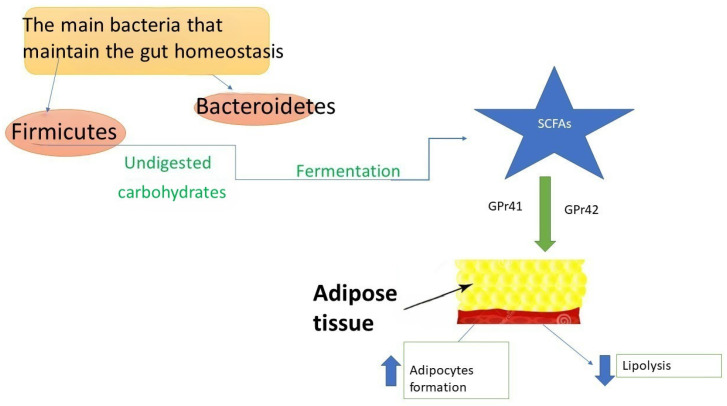
The main roles of short chain fatty acids (SCFAs) in pediatric obesity. Through G-protein receptor-41 and G-protein receptor-42 expressed at the adipocytes level (Gpr41 and GPr43), SCFAs interact with adipose tissue promoting adipocyte formation and inhibiting lipolysis.

SCFAs play a significant role in the physiopathology of obesity (Figure 1). They interact with adipose tissue through two G-protein-coupled receptors expressed on adipocytes (Gpr41 and Gpr43). This interaction promotes adipocyte differentiation while inhibiting lipolysis, thereby influencing fat storage and metabolism [43]. Moreover, they downregulate the hunger suppressing hormones such as glucagon-like peptide-1 (GLP-1), peptide YY (PYY), and leptin [44]. The gut microbiota can regulate appetite and satiety through immune-neuroendocrine mechanisms and vagus nerve activation [45]. Additionally, by activating the farnesoid X receptor, it can enhance bile acid metabolism, thereby influencing hepatic triglyceride levels and glucose homeostasis [46,47].

*Firmicutes* phyla play an important role in metabolism and nutrition because they synthesize SCFAs. The metabolic products of *Firmicutes* can influence hunger and satiety [9]. Most research suggests that *Firmicutes* bacteria have a greater capacity to ferment and metabolize carbohydrates and lipids, contributing significantly to obesity development [40]. In contrast, *Bacteroidetes*, a group of Gram-negative bacteria, play an essential role in immunomodulation [48]. The *Firmicutes*-to-*Bacteroidetes* (F/B) ratio is considered crucial for maintaining gut homeostasis, with studies reporting an increased F/B ratio in children with obesity [49,50].

A study comparing the gut microbiota of obese and lean individuals found that participants with obesity exhibited significantly lower levels of *Clostridium perfringens* and *Bacteroidetes* [51]. Another taxon, *Christensenellaceae* spp., has recently been linked to obesity and proposed as a new microbial biomarker, as it was shown to reduce weight gain in mice and alter gut microbiota composition [52]. Additionally, certain gut microbiota, like *Bacteroides thetaiotaomicron* alongside *Methanobrevibacter smithii*, were found to facilitate adipose tissue accumulation [53]. *A. muciniphila* is linked to improved metabolic health, enhancing glucose regulation, blood lipid levels, and body composition following calorie restriction in humans [54]. However, the relationship between *A. muciniphila* and improved metabolic health is currently associative, with causality not established. These findings support the idea that obesity is associated with an altered F/B ratio, characterized by increased *Actinobacteria* and decreased *Verrucomicrobia* [55]. A substantial body of research has examined associations between body mass index (BMI) and variations in gut microbial composition [56,57,58]. In particular, increased abundance of species such as *Prevotella copri* and *Bacteroides vulgatus* has been linked to insulin resistance through their involvement in the production of metabolites—including branched-chain amino acids, tryptophan derivatives, and LPS—that are implicated in metabolic disorders [59,60,61,62].

Moreover, the fungal microbiota or mycobiome was recently assessed in individuals with obesity versus those without obesity using an Internal Transcribed Spacer-based sequencing approach [63]. The study revealed that the mycobiome of individuals with obesity showed a higher prevalence of *Ascomycota*, specifically the class *Saccharomycetes*, as well as families *Dipodascaceae* and *Saccharomycetaceae*. There was also an increase in *Tremellomycetes* fungi compared to individuals without obesity, with *Mucor racemosus* and *Mucor fuscus* being more prevalent in patients without obesity. Notably, the relative abundance of the *Mucor* genus increased following weight loss in individuals with obesity, mirroring trends observed with *Bacteroidetes* [63].

In animal models, the cecal microbiota from obese mice, compared to lean mice, was found to be enriched in enzymes that break down nondigestible carbohydrates, leading to increased SCFA production and energy storage [30,64,65]. SCFAs contribute to obesity by providing an additional source of calories, though further research is needed in this area.

Differences in gut microbiota at the genus level and specific metabolites are more commonly linked to pediatric overweight status and obesity than differences at the phylum level. The impact of gut microbiota is largely mediated by the absorption and distribution of their metabolites [66]. These microbes produce a variety of metabolites that enter the bloodstream and can exert systemic effects on the host [67]. Recent studies have highlighted the association between obesity and metabolites such as amino acids, SCFAs, amines, medium-chain fatty acids, and bile acids. Distinctions in gut microbiota and metabolites between individuals with obesity and those of normal weight have been observed; for example, a reduction in *Bacteroides thetaiotaomicron*, which metabolizes glutamate, is linked to a higher obesity risk, while overweight adolescents display a greater capacity for carbohydrate oxidation [68]. These findings suggest that targeting gut microbiota and their metabolites could serve as a promising intervention for overweight and obese patients. Alterations in gut microbiota composition have been linked to pediatric obesity and non-alcoholic fatty liver disease. The biosynthesis of amino acids, SCFAs, and LPS is inversely correlated with insulin resistance, whereas pathways related to peptidoglycan biosynthesis exhibit a positive correlation with insulin resistance [62].

Propionate, a metabolite partially produced by *Clostridium* species, has been positively associated with pediatric overweight and obesity [69]. Moreover, higher fecal propionate levels have been correlated with elevated fasting blood glucose and glycosylated hemoglobin concentrations, as well as an increased risk of type 2 diabetes [70,71]. Children not exposed to human milk exhibit higher fecal propionate levels than breastfed children [72]. Additionally, higher serum propionate concentrations have been positively associated with BMI in children with obesity, while fecal propionate levels are significantly elevated in overweight and obese pediatric populations [73,74], findings that are consistent with observations reported in overweight adults [75]. Notably, lactate levels are lower in overweight children. As lactate serves as a substrate for propionate metabolism, this finding suggests that the gut microbiota may enhance propionate production [75,76].

Butyrate plays a crucial role in regulating energy metabolism and immune function. Key butyrate-producing bacteria include *Faecalibacterium prausnitzii* and *Roseburia hominis* [77], with *Faecalibacterium prausnitzii* being particularly important for maintaining gastrointestinal and metabolic health [75]. In children with obesity, a decline in butyrate-producing strains has been noted, and fecal butyrate levels have been inversely correlated with gut microbiota diversity. This reduction may contribute to altered intestinal permeability and metabolic dysfunction.

Butyrate exerts its effects through multiple metabolic pathways, including G-protein-coupled receptors GPR41 and GPR43, β-oxidation [78], and the inhibition of class I/II histone deacetylases. Additionally, butyrate stimulates the secretion of GLP-1, which enhances insulin sensitivity and plays a role in glucose homeostasis [79,80].

However, some studies conducted on humans and also on animal models, refs. [81,82,83] suggest that SCFAs may contribute to weight loss by reducing inflammation associated with obesity and metabolic disorders. These findings highlight the need for further research to fully elucidate the complex roles of these metabolites in metabolic health.

In conclusion, SCFAs exert context-dependent effects on adiposity and metabolic health. Under conditions of adequate dietary fiber intake, balanced energy consumption, and preserved microbial diversity, SCFAs—particularly butyrate and propionate—support metabolic homeostasis by strengthening gut barrier integrity, stimulating GLP-1 and PYY secretion via GPR41 and GPR43 activation, enhancing insulin sensitivity, promoting fatty acid oxidation, and modulating inflammation. In this context, they are generally considered to exert anti-obesity and metabolically protective effects [84,85,86,87]. In contrast, in the setting of dysbiosis, chronic overnutrition, and sustained positive energy balance, increased SCFA production may reflect enhanced microbial extraction of energy from indigestible substrates, thereby increasing total caloric availability to the host [11]. Moreover, experimental evidence suggests that excessive acetate exposure under hypercaloric conditions may stimulate insulin secretion and lipogenesis, potentially favoring adiposity [88]. Collectively, these findings indicate that SCFAs function primarily as metabolic regulators whose impact depends on dietary composition, microbial ecology, and host metabolic status rather than acting as inherently obesogenic or anti-obesity agents.

One recent study [89] emphasizes the growing importance of the oral microbiota and oral viral communities in the development and progression of pediatric obesity. The oral cavity represents a complex microbial ecosystem that may influence systemic metabolic processes through interactions with the gut microbiome and host immune responses. Alterations in microbial diversity and composition have been reported in children with overweight and obesity, suggesting that oral dysbiosis may contribute to metabolic imbalance and chronic low-grade inflammation. Therefore, the oral microbiome and virome are increasingly being investigated as potential biomarkers and therapeutic targets for understanding the mechanisms underlying pediatric obesity.

Recent research highlights the growing importance of the gut virome as a key component of the human microbiome involved in metabolic regulation. The gut virome is predominantly composed of bacteriophages that modulate bacterial populations and influence microbial ecosystem dynamics, thereby affecting host metabolic homeostasis [90]. Alterations in viral diversity and composition have been reported in metabolic disorders, including obesity and type 2 diabetes, suggesting that viral dysbiosis may contribute to metabolic dysfunction through disruption of phage–bacteria interactions and microbial community structure [91]. In pediatric populations, metagenomic analyses have demonstrated that children with obesity exhibit significant changes in gut double-stranded DNA (dsDNA) viral richness and diversity, which are associated with obesity and metabolic syndrome-related traits [92]. Because bacteriophages regulate bacterial taxa involved in energy metabolism, inflammation, and intestinal barrier function, alterations in the virome may contribute to microbiome dysbiosis and metabolic imbalance during childhood. These findings suggest that the gut virome represents an additional layer of microbial regulation and may serve as a potential biomarker or therapeutic target in pediatric obesity and related metabolic disorders.

## 4. Environmental Factors That Can Be Used to Improve Obesity-Related Microbiota Modifications

### 4.1. Dietary Patterns and Lifestyle Factors

Diet is a major determinant of gut microbial diversity and composition [30,64]. Reduced microbial diversity and alterations in specific bacterial taxa have been associated with metabolic disorders and obesity. Lifestyle interventions, including changes in dietary habits and physical activity, can modify gut microbiota composition. A pilot study in Mexican children with obesity evaluated microbiota changes following a six-week multidimensional lifestyle intervention consisting of a hypoenergetic diet combined with nutritional counseling and physical activity recommendations. Although waist circumference decreased, which was associated with increased *Odoribacter* abundance, overall microbiota diversity and composition remained largely unchanged [93].

Previous studies have linked *Odoribacter* abundance to visceral and subcutaneous adiposity, reporting higher levels in healthy individuals and lower levels in patients with obesity [94,95]. In a Korean study of 46 children, those with obesity exhibited lower relative abundance of Bacteroidetes and a significantly higher F/B ratio compared to normal-weight peers [96]. The study also highlighted the contribution of non-dietary factors, including cesarean delivery, reduced physical activity, increased screen time, and elevated metabolic and inflammatory biomarkers, emphasizing that obesity results from the interaction of microbiota, lifestyle, and environmental factors [97].

Experimental studies further support the role of diet–microbiota interactions in obesity. Ley et al. [8] demonstrated a reduced abundance of Bacteroidetes and increased Firmicutes in genetically obese (ob/ob) mice compared to lean controls, despite identical diets. Additionally, transplantation of microbiota from obese mice into germ-free mice resulted in greater fat accumulation [48]. In humans, O’Keefe et al. [97] showed that switching from a Western diet (high fat, low fiber) to a rural African diet (low fat, high fiber) for two weeks significantly improved mucosal inflammation and metabolic markers.

Geographical dietary patterns also shape microbiota composition. Children from Burkina Faso consuming a fiber-rich, plant-based diet displayed a microbiota enriched in Bacteroidetes and *Prevotella*, along with higher abundance of *Treponema*, *Succinivibrio*, and *Weissella*, compared to Western children consuming diets high in animal protein, simple sugars, and fat [15,30,64]. Notably, *Treponema succinifaciens*, a carbohydrate-metabolizing species, may reflect specific dietary exposures such as termite consumption. These genera decline when individuals transition to urbanized environments. Similarly, a study in overweight Hispanic preschool children reported BMI reduction following a six-month behavioral intervention, though microbiota responses were highly individualized [98].

Paniz J. et al. [99] found that self-reported high screen time (≥75 min/day) was not associated with significant differences in overall gut microbial diversity (alpha and beta diversity) when accounting for BMI, age, sex, and physical activity, but specific bacterial taxa such as *Bacteroides*, *Prevotella*, and *Roseburia* showed differential abundance between high and low screen-time groups. Integrated analysis of microbiome and metabolome profiles suggested that high screen time is linked to distinct microbial and metabolic signatures that are hypothesized to relate to mitochondrial dysfunction, altered amino acid metabolism, and increased risk for metabolic disturbances including obesity and type 1 diabetes, providing a molecular framework for future investigation into lifestyle-mediated gut microbiome alterations.

### 4.2. Type of Foods and Specific Nutrients

A. Protein Intake:

High protein consumption influences microbial metabolism. Excess protein undergoes bacterial fermentation—primarily by *Bacteroides*, *Clostridium*, and Proteobacteria—producing metabolites such as phenols, ammonia, sulfides, amines, and SCFAs, which may affect host metabolic and systemic functions [100,101,102,103,104].

B. Fiber and Plant-Based Foods:

Dietary fiber promotes beneficial microbiota profiles. High fiber intake increases SCFA-producing bacteria, including *Actinobacteria* and *Bacteroidetes*, while reducing certain *Firmicutes* species [105]. Greater consumption of fruits, vegetables, nuts, and yogurt has shown to be negatively correlated with weight gain, whereas sugary beverages, potato chips, and red meat correlate positively with weight gain [106]. However, dietary quality often declines from childhood to adolescence, with reduced intake of fruits, vegetables, and dairy and increased protein consumption [107].

C. Dietary Fat

Over recent decades, shifts toward high-fat dietary patterns have paralleled increases in obesity and metabolic diseases. In China, the transition from a high-carbohydrate, low-fat diet to a lower-carbohydrate, higher-fat pattern has coincided with rising rates of obesity, type 2 diabetes, colon cancer, and cardiovascular disease [108]. High-fat diets—particularly those rich in long-chain saturated fatty acids—have been positively associated with obesity and may trigger inflammatory responses [109].

D. Carbohydrates and Sugars

Fructose consumption is of particular concern, as it may enhance de novo hepatic lipogenesis through acetate production by the gut microbiota [110,111,112]. The World Health Organization recommends limiting sugar intake to less than 25 g/day [113,114]. Although sugary beverage consumption has declined among children, these drinks remain a major source of caloric intake and are associated with obesity [115,116]. Beyond sugar, overall carbohydrate intake patterns are also relevant. While short-term carbohydrate restriction may improve body weight and glycemic control, long-term restriction may reduce fiber intake and increase fatigue, limiting sustainability. Further research is needed to evaluate long-term safety and efficacy in pediatric populations [117].

E. Coffee and Caffeine

Coffee consumption has been associated with protective effects against microbiota alterations induced by high-fat diets, including prevention of reductions in *Lactobacillus* and increases in *Bifidobacterium* [118]. Coffee intake may enhance microbial diversity and promote beneficial genera such as *Bifidobacterium* and *Akkermansia*. Additionally, chlorogenic acid, a coffee polyphenol, may stimulate SCFA production, supporting gut barrier integrity and metabolic health [119].

### 4.3. Physical Exercise

During the 2019 pandemic, the level of sedentary behavior among children rose significantly, leading to an increase in pediatric obesity prevalence [6,120]. Another study has shown that bacteria involved in the metabolism of proteins and fats are linked to weight gain, while those that metabolize fiber are associated with weight loss [121]. Key factors contributing to childhood obesity include diet, a sedentary lifestyle, and genetic predisposition. These factors together influence both the composition of the gut microbiome and the risk of developing obesity.

Children who had performed 12 weeks of physical activity showed no changes to their BMI, but they had decreased blood glucose and cholesterol concentrations. Thus, long-term interventions could have a better impact on overweight children’s health. Physical exercise was associated in many studies [122,123,124] with an increase in butyrate-producing bacteria (e.g., *Roseburia hominis*, *Faecalibacterium pausnitzii*, and *Ruminococcaceae*). More studies should be conducted in this field, because no consistent evidence of exercise impact on microbiota has been found.

A higher taxonomic diversity in rugby athletes was reported by Clarke et al. [125], and Allen et al. [126] reported that 6 weeks of endurance exercise training was associated with an increase in the microbiome alpha-diversity. Certain fecal SCFA levels and specific bacterial taxa were found to increase after physical training. However, when subjects reverted to a sedentary lifestyle, these concentrations gradually decreased until they returned to baseline levels. This suggests that regular physical activity may enhance gut microbiota composition and metabolic byproducts, but these beneficial changes are lost when sedentary behavior resumes.

Studies investigating the impact of exercise on the gut microbiota of both, human and animal models, have yielded mixed results. Some research has shown that exercise can reduce the abundance of *Firmicutes* and/or increase the levels of *Bacteroidetes* in the gut [127,128,129,130,131]. However, other studies have reported the opposite effect, with increases in *Firmicutes* and decreases in *Bacteroidetes* [132,133,134,135,136,137], while some research has found no significant impact of exercise on these bacterial populations [138,139,140,141,142,143,144]. These discrepancies may be due to differences in study designs, exercise regimens, or individual variations in microbiota composition. Regarding microbial diversity, some studies show increase in response to exercise [98,99,100,101,102,103,104,105,106,136,137], others decrease [126,127], and some detecting no change [129,138,139,140,144,145,146,147,148]. A previous study [129] found that exercise helped mitigate the reduction in alpha diversity observed in mice that were fed a high-fat diet. This suggests that physical activity may have a protective effect on gut microbiota diversity, even in the context of an unhealthy diet, potentially contributing to improved metabolic health and resilience against the negative impacts of a high-fat diet. Increased microbial diversity is often associated with better overall gut health and metabolic function.

Bacterial taxa that have been documented to respond to exercise training include *Bifidobacterium* (typically increased) [134,139,140], *Lactobacillus* (typically increased) [132,134,135,149], *Akkermansia* (typically increased) [139,140,142,143], and *Streptococcus* (variable effects) [135,138]. Exercise has also been associated with a decrease in *Proteobacteria* [134,135,139,150,151], *Turicibacter* [128,144], and *Rikenellaceae* [127,138,145]. The effects on *Clostridium* appear to be variable, with some studies reporting increases and others decreases [134,145,146,149,150]. Additionally, exercise has been linked to changes in measures of alpha- and beta-diversity, although these effects are variable across studies [127,128,135,139,141,152].

The multidimensional interventions which included physical activity led to very different results. For example, during a 6-week study no changes in the gut composition and diversity were observed [153], while in another study also carried out across 6 weeks, children classified as overweight who performed moderate to high-intensity exercise and had a calorie restricted diet were found to have a significant increase in their gut microbiota alpha diversity [154]. Moreover, researchers found a connection between physical exercise and improved alpha diversity [155]. During an 8 weeks weight loss program, gut bacterial alpha diversity was found to be lower, while beta-diversity showed no difference in Korean children affected by obesity [156]. Furthermore, intense exercise training led to a reduction in the F/B ratio. This shift in the gut microbiota composition is significant, as a lower F/B ratio is often associated with improved metabolic health and weight regulation. This suggests that intense exercise may promote a healthier microbiome balance, which can contribute to overall well-being, especially in the context of weight management and metabolic function [154]. At the species level, one study [93] reported an increase in *Bacteroides fragilis* and a decrease in *Clostridium coccoides*, *Bifidobacterium adolescents*, and *Bifidobacterium longum*, after 10 weeks of regular physical exercise and an energy restricted diet. At the genus level, a lower abundance of *Streptococcus* [157] and *Bacteroides* [155], but higher levels of *Blautia*, *Roseburia*, and *Dialister* were found after the intervention [158]. In addition, a significant reduction in the proportion of Gram-positive bacteria (e.g., *Clostridium histolyticum* and *coccoides*, *Eubacterium rectale*), was associated with weight loss, and a significant increase in Gram-negative bacteria (e.g., *Bacterioides*, *Prevotella*) in participants with >4 kg of weight loss was found in adolescents who participated in a multidisciplinary obesity treatment program [8].

There is evidence suggesting that bariatric surgery can partially reverse obesity-related microbial changes and contribute to weight loss. Studies [62,159,160] have demonstrated that this surgical intervention results in a significant alteration in the gut microbiota, leading to improved metabolic outcomes. Previous research on Hispanic, Mexican, and Korean children have already revealed notable differences in the gut microbiota composition between children with and without obesity [96,99,161]. In a prospective study, Rampelli S. et al. [161] investigated microbial shifts in individuals who experienced weight gain over a four-year period and concluded that alterations in gut microbiota composition could serve as a potential predictor for obesity. These findings underscore the significant role of gut microbiota in weight management and the development of obesity [162,163].

## 5. Obesogenic Microbiota

Many studies have endeavored to identify bacteria species that have a crucial role in the development of obesity. The most common finding in the microbiota composition was an increased abundance of opportunistic pathogens and a reduction in butyrate producing microbes. Families *Christensenellaceae* and *Rikenellaceae*, as well as genera *Oscillospira*, *Bifidobacterium*, and *Akkermansia* were all found to be decreased in abundance [163]. *Akkermansia muciniphila* is widely recognized as a beneficial bacterium in the context of obesity and related metabolic disorders. Its presence is associated with improved metabolic health, and it is often found in lower amounts in individuals with obesity [36]. Research has also indicated that overweight children tend to have a lower proportion of *Bifidobacterium* in their gut during early infancy compared to their normal-weight counterparts [164]. Additionally, the levels of *Lactobacillus gasseri* and *Lactobacillus reuteri* have been positively correlated with obesity, while *Lactobacillus paracasei* shows a negative correlation, suggesting that not all Lactobacillus species have the same impact on body weight [165]. These findings highlight the complex role of gut microbiota in obesity and suggest specific bacterial species that could be targeted for therapeutic interventions (Table 1).

A systematic review concluded that the gut microbiota of individuals with obesity tends to be enriched in *Lactobacillus* and *Proteobacteria*, while it shows a reduction in *Bifidobacterium*, *Bacteroidetes*, and overall alpha diversity [166]. This reduced microbial diversity is often linked to metabolic dysfunctions and obesity. On the other hand, physical activity has been shown to enhance alpha diversity, which is associated with healthier metabolic profiles and weight loss. These findings suggest that maintaining microbial diversity through lifestyle interventions like exercise may play a crucial role in managing obesity. *Firmicutes* phylum was more abundant in overweight children than in lean ones [167,168], but one study reported opposite findings [169]. Lower proportions of *Bacteroidetes* [95,167,170,171,172], *Actinobacteria* [169,173,174], *Akkermansia muciniphila*, *Candida* spp., *Faecalibacterium prausnitzii*, *Saccharomyces* spp. [175], *Bifidobacterium* spp. [174], *Bacteroides vulgatus* [176], and *Verrucomicrobia* [169,175] were found in the patients affected by obesity and those classified as overweight, while the proportion of *Proteobacteria phylum* and *Lactobacillus* was higher in the group of children affected by obesity [135,136,137], as well as the proportion of *Lachnospira* [134,142], *Actinomyces*, *Romboutsia*, *Weissella* [50], *Enterococcus*, *Sutterella*, *Klebsiella*, and *Collinsella* [141], *Blautia* [171,173], *Faecalibacterium* [172], and *Prevotella* [172]. It seems that there is a second mechanism in which the intestine’s microbiota is implicated in obesity: it disrupts the epithelial barrier integrity, leading to moderate systemic chronic inflammation [169]. Regarding the F/B ratio, several studies have documented a significant increase in children with obesity compared to their normal-weight counterparts [95,167,176,177]. Alterations in the relative abundance of major gut bacterial phyla—particularly *Firmicutes* and *Bacteroidetes*—have been implicated in inflammatory processes and obesity. Firmicutes (including genera such as *Clostridium*, *Enterococcus*, *Lactobacillus*, and *Ruminococcus*) and *Bacteroidetes* (comprising *Prevotella*, *Bacteroides*, *Parabacteroides*, and *Alistipes*) represent two dominant phyla within the human intestinal microbiota [178,179,180,181].

Several studies have reported differences in the F/B ratio between individuals with and without obesity. In a Japanese cohort, individuals with obesity exhibited a higher proportion of Firmicutes (40.8% vs. 37%) and a lower proportion of Bacteroidetes (37% vs. 44%) compared to lean participants [182]. Similarly, research in Ukrainian adults demonstrated a significant association between the F/B ratio and BMI, with individuals presenting an F/B ratio >1 showing a 23% increased likelihood of being overweight [183]. Consistent findings were observed in Qatari and Kazakh populations, where the F/B ratio was elevated in individuals with obesity relative to lean controls [48,184]. Collectively, these data support an association between shifts in gut microbial composition and obesity-related metabolic disturbances.

The systematic review by Ejtahed et al. [185] examined human studies investigating gut microbiota-derived metabolites in overweight and obese individuals and reported consistent alterations in circulating branched-chain and aromatic amino acids, glutamate, SCFAs, bile acids, and choline- and carnitine-related metabolites. These metabolic shifts were associated with insulin resistance, dyslipidemia, inflammation, and cardiovascular risk, highlighting the mechanistic role of microbial metabolites as mediators linking gut dysbiosis with metabolic dysfunction. Similarly, the review published by Koumpouli et al. [186] emphasized the modulatory role of dietary patterns and functional foods—such as probiotics, prebiotics, polyphenol-rich products, and adherence to the Mediterranean diet—on gut microbiota composition and metabolic outcomes. The authors reported that fiber- and polyphenol-rich diets promote beneficial taxa and SCFA production, reduce pro-inflammatory species, and may improve insulin sensitivity and metabolic syndrome parameters, underscoring diet–microbiome interactions as a therapeutic target.

Finally, the seminal review “*The gut flora as a forgotten organ*” [187] conceptualized the intestinal microbiota as a metabolically active organ that contributes to energy harvest, immune modulation, vitamin synthesis, and protection against pathogens. It proposed that alterations in microbial composition can influence host energy balance and lipid metabolism, providing early theoretical support for the role of gut microbiota in obesity and metabolic diseases.

## 6. Treatment That Can Be Used in Obesity-Related Microbiota Alterations

### 6.1. Probiotics

Probiotics, as defined by WHO, are live microorganisms that can confer health benefits to the host when administered in adequate amounts [188]. These microorganisms have been shown to exhibit potential anti-obesity and anti-inflammatory properties, which makes them an area of interest in managing obesity-related conditions. However, while preliminary studies suggest promising effects, more extensive research is needed to fully understand their impact on obesity and inflammation and to determine effective strains, dosages, and mechanisms [189,190].

Probiotic administration could be used to modify microbiota composition and to prevent and treat some of the pediatric diseases like atopic diseases, obesity, and inflammatory disease. Several studies have investigated the potential of probiotics, prebiotics, and synbiotics to promote weight loss and improve metabolic health [14,191,192,193,194,195]. One such study involving adults found that modifying the composition of the gut microbiota through probiotic intake was associated with a reduction in body weight [192]. Additionally, another study demonstrated that supplementation with *Akkermansia muciniphila* led to improvements in metabolic parameters in overweight individuals, highlighting its potential role in weight management and metabolic health [192]. A total of 120 infants were studied following supplementation with *Lactobacillus paracasei* F19 throughout the weaning period. This treatment had no impact on body weight at school age [193]. A study examining the effects of a probiotic/prebiotic mixture in children affected by obesity revealed promising results, showing a decrease in body fat along with an increase in *Bifidobacterium* species within the gut microbiota [194]. In addition, two more studies demonstrated a reduction in BMI z-score after 8 weeks of symbiotic administration to overweight children [14,195,196].

Probiotics are emerging as a promising intervention for reducing the F/B ratio, a microbial marker often associated with obesity. Beneficial bacteria such as *Bacillus*, *Lactobacillus*, and yeasts from the *Saccharomyces* genus have been the focus of research, examining their potential to improve gut microbiota composition and lower obesity risk.

Studies have shown that administering *Lactobacillus sakei* and *Lactobacillus rhamnosus* GG effectively reduced the F/B ratio in obese mice [197]. Furthermore, when *L. rhamnosus* GG was consumed alongside a high-fat diet, it not only decreased the F/B ratio but also prevented weight gain in a murine model [198]. In another study, hyperlipidemic rats treated with *L. rhamnosus* exhibited reduced serum lipid levels after 56 days of treatment [199]. Additionally, a 12-week treatment with *Lactobacillus paracasei* and xylooligosaccharide improved insulin sensitivity, reduced body weight, lowered LDL cholesterol levels, and decreased the F/B ratio [200]. Probiotics from fermented milk were administered to healthy and overweight adults and significant decreases in visceral and subcutaneous fat, body weight, and BMI were found [46].

Another probiotic, *Bacillus amyloquefaciens*, has also shown potential in reducing body weight, F/B ratios, and hepatic steatosis in mice fed with a high-fat diet [201]. Similarly, *Saccharomyces boulardii* was administered to type 2 diabetic and leptin-resistant obese mice over four weeks. This treatment resulted in reduced body weight, a lower F/B ratio, and decreased hepatic steatosis [202].

Several strains, including *Lactobacillus rhamnosus* GG, *Lactobacillus gasseri*, and *Bifidobacterium breve*, have been explored for their potential effects on BMI, adiposity, and metabolic parameters, with some studies reporting modest improvements in weight regulation, insulin sensitivity, and inflammatory markers [203,204]. However, findings remain inconsistent due to substantial heterogeneity in study design, small sample sizes, short intervention periods, strain-specific variability, differences in dosage and formulation, and limited long-term follow-up. Moreover, pediatric populations present additional challenges, including developmental variability in microbiota composition and ethical considerations in conducting long-term randomized trials. Therefore, while probiotic supplementation represents a promising adjunct within multidimensional lifestyle interventions, larger, well-controlled, and longitudinal studies are necessary to clarify strain-specific efficacy, optimal dosing strategies, safety profiles, and durability of metabolic effects before routine clinical implementation can be recommended.

### 6.2. Fecal Microbiota Transplantation

Another way to modify the intestine microbiota is fecal microbiota transplantation (FMT). This method has already been investigated in several studies [205,206]. The first one [205], used feces from a vegan donor and transferred them to the patients with obesity. Its results showed no changes in their BMI. The second one [206] demonstrated that the microbiota composition after FMT via oral capsules from a single lean donor intake was changed, but there were also no significant changes in BMI. Karen S. et al. [207] conducted a randomized, placebo-controlled trial including 87 adolescents with obesity and reported that FMT from lean donors did not significantly reduce BMI at 6 weeks, indicating that FMT alone is not an effective weight-loss strategy in this population. Nevertheless, FMT was associated with a sustained decrease in the android-to-gynoid fat ratio for up to 26 weeks—particularly in female participants—and induced transient but measurable shifts in gut microbiome composition. Exploratory analyses also suggested a higher rate of metabolic syndrome resolution among participants with previously unrecognized metabolic syndrome at baseline. The intervention was well tolerated, with no serious adverse events reported.

In a subsequent four-year follow-up of the same double-blind, randomized trial, no significant differences in BMI were observed between FMT and placebo groups after adjustment for confounding factors. However, adolescents who received FMT exhibited long-term improvements in body composition and metabolic health, including reduced waist circumference and total body fat percentage, lower metabolic syndrome severity scores and hs-CRP levels, and increased HDL cholesterol, while glucose metabolism and other lipid parameters remained unchanged. Metagenomic analyses demonstrated persistent alterations in microbial diversity, functional capacity, and stable engraftment of donor-derived bacterial strains and bacteriophages. Collectively, these findings indicate that although FMT does not promote weight loss in adolescents with obesity, it may induce durable improvements in metabolic risk markers and gut microbiome structure [208].

## 7. New Strategies for Diverse Bacterial Species Identification

Next-generation sequencing (NGS) offers several key advantages over traditional culture methods, particularly in its capacity to detect a broader range of unique microbial species. One of the major strengths of NGS is its ability to sequence multiple samples simultaneously, allowing for high-throughput analysis. Furthermore, it can directly identify microbial DNA or RNA from various types of biological samples, including tissue, fecal matter, or blood, making it a versatile tool in microbial and metagenomic research [171,209,210]. This technology has significantly advanced our understanding of complex microbial communities and their roles in human health, providing insights that were previously unattainable through culture-based techniques. NGS could be used to predict disease risk by exploring the human genome, but more research needs to be conducted in this context. Using 16S rRNA gene sequencing, Xiaowei Chen et al. [171] analyzed 30 normal-weight, 35 overweight, and 35 obese patients from China. They concluded that microbiota alpha diversity decreased and, regarding the beta-diversity, significant differences between these three groups were noted. The number of microbiota species that were changed was also different: 31 species in normal-weight, 32 in group with obesity and 3 in overweight patients. In the same study, alpha diversity metrics showed a significant decline in species richness with increasing BMI, as reflected by indices including Chao1 (F = 5.478, *p* = 0.006), observed species (F = 7.271, *p* = 0.001), and PD whole tree (F = 8.735, *p* < 0.001), indicating that gut microbial species diversity decreased significantly as BMI increased.

## 8. Conclusions

Although accumulating data indicate a strong link between gut microbiota imbalance and pediatric obesity, existing studies are constrained by methodological variability, limited cohorts, and insufficient follow-up durations. Future research should aim to elucidate causal mechanisms, particularly the impact of early-life microbial colonization on subsequent obesity risk, and to define actionable microbial and metabolic targets for personalized interventions. Large, rigorously designed longitudinal trials—especially those evaluating the long-term safety and effectiveness of probiotic and symbiotic strategies—are necessary to support clinical translation. Importantly, the rising prevalence of childhood obesity cannot be attributed to genetic predisposition alone. Environmental influences, dietary patterns, and physical inactivity substantially contribute to disease development. Diets high in refined sugars, fats, and protein but poor in fiber, combined with sedentary behavior, promote intestinal dysbiosis and the establishment of an “obesogenic” microbiota. This altered microbial community enhances the fermentation of undigested carbohydrates, increasing SCFA production, which may influence adipogenesis, lipid metabolism, and energy balance. Evidence suggests that lifestyle optimization—including balanced nutrition and regular physical activity—can restore microbial diversity and improve metabolic outcomes. While adjunctive probiotic supplementation has shown encouraging results when integrated into multidimensional interventions, additional high-quality evidence is required before microbiota-targeted therapies can be routinely implemented in the management of pediatric obesity.

## Figures and Tables

**Table 1:** Gut microbiota characteristic species in overweight and obese children.

Obesogenic Microbiota Abundance	Genera
↑ (increased abundance)	*Lactobacillus reuteri* [123]
	*Lactobacillus gasseri* [123]
	*Proteobacteria* [124]
	*Firmicutes* [125,126]
	*Lachnospira* [129,136]
	*Actinomyces* [55]
	*Romboutsia* [55]
	*Weissella* [55]
	*Enterococcus* [135]
	*Sutterella* [135]
	*Klebsiella* [135]
	*Collinsella* [135]
	*Blautia* [129,135]
	*Faecalibacterium* [128]
	*Prevotella* [130]
↓ (decreased abundance)	*Rikenellaceae* [120]
	*Christensenellaceae* [120]
	*Bifidobacterium* [120]
	*Oscillospira* [120]
	*Akkermansia muciniphila* [120]
	*Bacteroidetes* [50,125,128,129,130]
	*Actinobacteria* [127,131,132]
	*Candida* spp. [133]
	*Faecalibacterium prausnitzii* [133]
	*Saccharomyces* spp. [133]
	*Bacteroides vulgatus* [134]
	*Verrucomicrobia* [127,135]

## Data Availability

No new data were created or analyzed in this study. Data sharing is not applicable to this article.
